# Service-Related Musculoskeletal Injuries in Polish Firefighters: A 2021–2023 Accidents Analysis

**DOI:** 10.3390/healthcare13060684

**Published:** 2025-03-20

**Authors:** Łukasz Dudziński, Łukasz Czyżewski, Janusz Wyzgał

**Affiliations:** 1 Medical Resuce Department, Medical University of Warsaw, Żwirki i Wigury 61, 02-091 Warsaw, Poland; 2 Department of Geriatric Nursing, Medical University of Warsaw, Żwirki i Wigury 61, 02-091 Warsaw, Poland; czyzewski_lukasz@wp.pl; 3 Department of Nephrology Nursing, Medical University of Warsaw, Żwirki i Wigury 61, 02-091 Warsaw, Poland; janusz.wyzgal@wum.edu.pl

**Keywords:** musculoskeletal injuries, ankle injury, firefighter health, health hazards, fracture, sprain

## Abstract

**Aim:** The aim of this study was to analyze service-related musculoskeletal injuries of professional firefighters in 2021–2023. **Material and methods:** Analysis was completed on the basis of annual reports on the injury status of the State Fire Service (SFS) obtained from the Office of Occupational Safety and Health (OOSH) at General Headquarters. The report databases were searched using keywords typical of bone injuries and including anatomical names: “sprain”, “fracture”, “dislocation”, “bone injury”, “bone”, “joint”, “spine”, “skull and “musculoskeletal injury”. **Results:** Events matching the analysis target of N = 1944 (2021—n = 707; 2022—n = 589; 2023—n = 648) accounted for 49.4% from all accidents in the State Fire Service in Poland in the analyzed period. A significant increase in the analyzed period 2021–2023 was observed in events where the cause of injury was sports activities (45% vs. 49% vs. 63%, *p* < 0.001). A significant decrease was observed in events where the cause of injury was interventions (26% vs. 27% vs. 17%, *p* < 0.001), technical and maintenance (TM) work (8% vs. 6% vs. 5%, *p* = 0.008) and category “other” (15% vs. 14% vs. 11%, *p* = 0.034). Statistically significant differences were found between cause and the type of injury (*p* = 0.002), season (*p* < 0.001) and the location of injury (*p* < 0.001). **Conclusions:** A large number of musculoskeletal injuries are associated with sports activities, which is inherent in the risks of the activities. The lower extremities are most affected by injuries during sports activities The spring period dominates in the season category, which may be related to the difficulty of the terrain and return to increased sports activity after the winter period.

## 1. Introduction

Health risks in the practice of professional firefighters can be divided into injuries caused by a sudden external factor with a dynamic course or occupational diseases that develop over a long period of time caused by constant risk factors characteristic of the profession. Firefighters’ injuries encompass a range of accidents that can arise from unforeseen events from exposure to harmful substances and psychosocial responses to adverse labor conditions and to repetitive movements. The range of threats for firefighters is wide due to the dynamic and emergency nature of the intervention. There are also office positions in the fire brigade, and these firefighters may also experience injuries [1,2].

Risk factors and an unfavorable variable microclimate generate numerous overloads on the firefighter’s body. Variability is associated with a variety of interventions, e.g., extreme temperatures and humidity (hot air—fire, excessive moisture—during disaster recovery and natural disasters). Risks to the health of firefighters are caused by physical and chemical factors, physical strain, unstable ground and the dynamics of the event combined with time pressure to complete the task. Air pollution, radiation and biological factors are other groups that have a negative impact on firefighters’ health [3,4].

Firefighters’ injuries occur despite the use of personal protective equipment during interventions and exercises. Protective equipment, including special clothing, boots, a helmet and gloves protect all areas of the body; in addition, firefighters use respiratory protection at certain hazards. A common result of injuries in the practice of firefighting is injuries in the area of bones and bone connections. A firefighter’s skeletal system is exposed to many occupational stresses, which include impacts, falls due to slips, trips, falling objects, lifting and transporting objects of considerable weight (tools, equipment) and carrying casualties as part of an evacuation [5,6].

There are other tasks in a firefighter’s duty cycle other than rescue and firefighting interventions; in which case, firefighters are not as protected as they are during operations. Other scheduled activities of firefighters according to the duty schedule include drills, training, sports practices, sports competitions, equipment demonstrations, annual fitness tests, cleaning and maintenance of equipment and the inspection of rescue equipment and tools [7,8].

Numerous studies point to musculoskeletal injuries and strains as a hazard for firefighters. Skeletal injuries result in long periods of firefighter indisposition, the need for replacements, increased medical costs and the risk of permanent damage to health. Among the areas of the body in the category of bone injuries, injuries to the back, knee joint, ankle joint and shoulder predominate. Risk factors for bone injuries include difficult, uneven, slippery ground (roofs, ladders and stairs) and limited visibility (night time and smoke). Factors referred to as the physical dynamic load are a loss of balance, falling, slipping, and work involving large muscle groups. These risks are associated with physical work during interventions: carrying loads, equipment, people, hitting immovable objects, dynamic force, crushing, collapsing ceilings and working with equipment (pneumatic and electric). Authors of studies in the field of firefighter health prevention also describe risk factors related to zoonotic type: the abnormal behavior of people and animals and the firefighter’s own carelessness and inattention [9,10,11,12].

The load on the skeletal system of firefighters is also affected by sports activities: training and competitions. Firefighters, as a formation that should excel in physical fitness, take part in many sports representing their units. Often firefighters have the status of professional athletes. Skeletal injuries as a result of sports are mainly team sports (contact with another player) and firefighting sports competitions that simulate real-life activities [13,14,15].

Sport is an important part of Polish firefighters’ duty. The calendar of sports activities is described in normative acts as mandatory activities. Firefighters taking part in sports competitions are considered to be on duty only when they represent their organizational unit and are delegated on the basis of relevant regulations and documents. A firefighter’s participation in competitions when representing his own service unit is included in the standard duty time [16].

The main aim of this study was to analyze service-related musculoskeletal injuries of professional firefighters in 2021–2023. The intermediate goal is to identify the causes, circumstances and frequency of events leading to injuries of the skeletal system in the practice of professional firefighters. The aim of the study is justified by the international literature, which shows that this type of injury is very common despite the use of numerous protective measures, procedures and training in the field of occupational safety [17,18,19]. This type of injury occurs in other professions [20,21,22].

## 2. Material and Methods

### 2.1. Research Design

This analysis is a cross-sectional descriptive study. Analysis was completed on the basis of annual reports on the injury status of the State Fire Service (SFS) obtained from the Office of Occupational Safety and Health (OOSH) at General Headquarters.

### 2.2. Research Setting

The survey included data from the OOSH. Data were collected from all over Poland in the form of an annual analysis of the injury status. The flow of information was from city and district commands through provincial commands to the office at the National Fire Service Headquarters. From reports including quantitative data, total number of injuries, number of injured firefighters, age categories of the injured, circumstances of the accident, injuries (area) of the body, classification of injuries into individual and collective and events matching the purpose of the analysis were selected. The report databases were searched using keywords typical of bone injuries and including anatomical names: “sprain”, “fracture”, “dislocation”, “bone injury”, “musculoskeletal injury”, “bone”, “joint”, “spine” and “skull”.

The data obtained from the search was divided into 3 categories for statistical calculations and correlations. Within each category, several detailed subcategories were extracted:

1. Type of activity: sports, rescue and firefighting, drills/training, technical and maintenance (TM) work and other (moving between buildings and falling on stairs);
2. Type of injury: sprain, fracture, laceration and other type;
3. Body area: lower limb, upper limb, chest, torso and head.

The observations focused on SFS officers and their tasks arising from the course of duty. The population covered by the observation is all officers (except civilian employees) regardless of the type and location of their service. The structure of the National Rescue and Firefighting System (NRFS) is made up of the General Headquarters, Provincial Headquarters, City Headquarters and County Headquarters in which Rescue and Firefighting Units (RFUs) as well as Specialist Rescue Groups (SRGs) operate. In these entities, firefighters are on duty on an 8 h and 24 h basis (shift duty). In addition, the potential of the SFS is supplemented by fire schools, the Central Museum of Fire Fighting, and the Scientific and Research Center for Fire Protection. Some units (museum and research institute) do not carry out rescue and firefighting interventions, but these units are staffed by professional firefighters, among whom there are also injuries that qualify for inclusion in our analysis [23,24].

This study was descriptive and retrospective in nature. The data were made available to the authors in the form of an electronic database in three Excel files from the Ms Office package. Each file covered the next year of analysis (2021, 2022 and 2023). Among all health risks described, the authors searched for records that met the inclusion criteria and then copied the record to the working database file.

### 2.3. Ethical Considerations

In October 2022, the Chief Fire Officer of SFS gave permission for access to the reports. The cases described are fully anonymous, the analysis complies with the principles of the Declaration of Helsinki, and the General Data Protection Regulation (GDPR), so the authors did not seek the consent and opinion of the bioethics committee. In Poland, the qualification of scientific research is specified in the Act on the professions of medical doctor and dentist of 5 December 1996 (*Journal of Laws* No. 97, No. 28, item 152). In Chapter 4, entitled “Medical experiment”, Article 21.1 describes the qualification of a medical experiment that is carried out on humans and may be a therapeutic or research experiment. In other cases, including where the work involves only a retrospective analysis of documentation, the consent of the bioethics committee does not apply [25].

### 2.4. Exclusion Criteria

Three areas are excluded from the analysis:

1. Population under observation: firefighters of Volunteer Fire Departments (VFDs) and civilian employees of SFS (these groups do not perform national service);
2. Time of observation: events occurring outside the date range of 1 January 2021–31 December 2023 were rejected. The date of injury (health hazard) was important, not the time of treatment and preparation of post-injury documentation;
3. The cause and circumstances of the injury—causing temporary indisposition of firefighters arising during off-duty time—were not included. Importantly, according to the rules of the course of duty, while on the way to and from activities, it is treated as an accident at work. In the analysis, there were several events (skeletal injuries during the way “to activities” and “return from activities”, classified in category 1—subcategory “other”);
4. Analysis does not include soft tissue injuries, visual injuries, thermal injuries, or hearing (acoustic) injuries.

### 2.5. Statistical Analysis

The database was prepared in Microsoft Excel using MS Office 2024 for Windows. Descriptive statistics were used to characterize the variables. The Kolmogorov–Smirnov test was used to assess the normality of the distributions. Qualitative variables (cause of interventions, injury type and location and season) were presented as quantity (n) and percentage values of the whole group (%), while proportions in groups were assessed with a Chi-square test. The *p*-values reflect differences within years of observation and cause of injury as independent variables. Missing data as a missing completely at random (MCAR) handling was carried out by complete-case analysis (CCA) and affected 0.7% of results. Sample size calculation: confidence level 95%, maximum error 4%. In the comparative analysis, Statistica 13.0 (StatSoft, Tulsa, OK, USA) was used for statistical analysis. *p* < 0.05 was considered as the significance level.

The analysis is retrospective. The cases included in the analysis are complete and reported to the appropriate OOSH. All cases are also medically confirmed and do not result from the subjective assessment of the firefighter. Each accident record created in the OOSH department is based on the firefighter’s medical documentation, which is later assessed by the appointed medical commission. Interrater agreement was not assessed. Reports that described the location of injury, the mechanism of injury and circumstances of injury did not leave room for different interpretations and were unambiguous.

## 3. Results

During the years of observation, 3934 service-related firefighter health risks occurred within the SFS (2021—n = 1312; 2022—n = 1266; 2023—n = 1356). Firefighter injury reports that fit the purpose of the analysis accounted for 49.4% of all duty accidents, n total = 1944 (2021—n = 707; 2022—n = 589; 2023—n = 648) (data in Table 1). The causes of this type of injury are mainly sports activities (contact with the opponent in team sports), dynamic load, rescue and firefighting activities and difficult terrain. The location of the injury is mainly the area of the knee joint, ankle joint, elbow or wrist. The most common effects of injury are joint sprain, joint tear and long bone fracture. The analysis of data (2021 vs. 2022 vs. 2023) covering the entire country showed no statistically significant difference in the incidence of injuries in relation to both the rate (‰) of all people employed in the fire brigade (*p* = 0.302) and the rate (‰) of interventions per year (*p* = 0.079). The observed years showed a statistically significant difference in the cause of injury (for the whole model, *p* < 0.001). A significant increase was observed in events where the cause of injury was sports activities (45% vs. 49% vs. 63%, *p* < 0.001). A significant decrease was observed in events where the cause of injury was interventions (26% vs. 27% vs. 17%, *p* < 0.001), TM work (8% vs. 6% vs. 5%, *p* = 0.008) and category “other” (15% vs. 14% vs. 11%, *p* = 0.034). It was also shown that the characteristics of the types of injury (for the whole model, *p* = 0.012) and the location of the injury (for the whole model, *p* < 0.001) were statistically significantly different in the observed years. A significant decrease was observed in events where the injury location was body—back (6% vs. 5% vs. 2%, *p* = 0.003). The *p*-values reflect differences within years of observation (2021 vs. 2022 vs. 2023)—Table 1.

Statistically significant differences were found between the type of injury (for the whole model *p* = 0.002), season (for the whole model *p* < 0.001) and the location of injury (for the whole model *p* < 0.001). Clinically significant differences were demonstrated for fractures most often related to TM work (24%, *p* = 0.115) and sprains to rescue and firefighting activities (62%, *p* = 0.168). Statistical differences between types of injuries were showed for tears (*p* < 0.001) was related to sport (N = 57; 6%). In summer, the most common injuries occurred during sports activities (33%, N = 339, *p* < 0.001), while in winter, rescue and firefighting activities (26%, N = 119, *p* < 0.001) and while in spring, exercise (36%, N = 34, *p* = 0.017). During sports activities, injuries to the lower extremities were most common (83%, N = 838, *p* < 0.001). The *p*-values reflect differences within cause of injury (Intervention vs. Sport vs. Other vs. Training vs. TM work)—Table 2.

## 4. Discussion

Firefighters, before being accepted for service, are selected for their health. They demonstrate sufficient condition and physical fitness to complete fitness tests successfully; in addition, they are examined by a number of medical specialists. Nowak et al. studied the relationship between the physical fitness of a selected group of firefighters in terms of anthropometric parameters, additional physical activity and the frequency of injuries. Using a battery of physical fitness tests, they determined the correlation between physical fitness and age, BMI and additional physical activity, as well as injury frequency. In our study, injury reports did not make firefighters’ BMI available [26].

The BMI of Polish firefighters was determined in another study from 2024. Observations of the heart rate variability (HRV) of firefighters subjected to psychophysical loads in a smoke chamber (a test in firefighters’ practice) showed that the average BMI of the firefighters included in the study was 25.3 with a mean age of 27, which is slightly above the normal weight range for the group. Being overweight may predict a greater tendency for bone injuries in overloads, but an analysis of weight and the resulting consequences was not the purpose of that study [27].

Nazari describes that the numerous stresses and demands of firefighters cause acute, recurrent or chronic pain complications. Based on a questionnaire, the main bone complaints of firefighters were identified: spine, lower extremity and upper extremity, more than one painful site and recurrent pain after injury [28].

The main types of injuries in our analysis were sprains involving the joints of the lower extremities: the ankle and knee. The literature on occupational injuries provides information that confirms our observations in the population of firefighters and other occupations with the nature of emergency work. Games points to slips, trips and falls as the main causes of musculoskeletal injuries in firefighters. Researchers hypothesized that heat stress is a major factor in amplifying injuries at the fire scene by negatively affecting measurements of functional balance [29].

The results of Ras et al. confirm that the incidence of work-related musculoskeletal injuries is noticeably high among firefighters, adding to the dangers of the job and the difficulties firefighters face on a daily basis. In this survey (author’s questionnaire), 73% of firefighters reported experiencing discomfort of moderate severity 1–2 times a week. The most common causes of injury were falls, jumps, slips and trips (39.7%) [30].

Lanham et al. state that exertion injuries happen to firefighters quite often, but, to increase work readiness and reduce the risk of injury, firefighters should exercise regularly. It is important to identify the mechanisms of injury and, in response to the causes, determine appropriate countermeasures [31].

Evdokimov conducted an analysis of firefighter injuries based on ICD-10 code classifications. In the reports, in the self-analysis, codes were available for n = 25 reports and were for ankle and knee injuries. The presentation of these results was waived due to the small number of data, as described in the Limitations section [32].

Eastman identifies risk factors and mechanisms associated with firefighter musculoskeletal injuries based on firefighter feedback. Observations were made of exertion-related injuries during training and did not include participation in interventions. Risk factors were identified: age, immobility, mobility and fatigue-related factors, and the results can be helpful in developing an exercise program, using movement assessment. This is important in our evaluation as well, since Polish firefighters have exercise written into their service as a mandatory point of duty to show increased fitness and capacity despite sports injuries [33].

Joint and lower-extremity injuries are prevalent not only in the firefighter population. Aksu et al. point out the strain on these body areas in the population of professional dancers. Lower limbs, especially the knee area (meniscus damage and anterior cruciate ligament damage), are injured as a result of prolonged hard landings and the impacts of overloading [34].

Two other studies look at knee loads in firefighter practice. Zhang noted the frequent occurrence of knee injuries during firefighter training [35]. Wang examined the forces on the firefighter’s body while working with respiratory protection apparatus (extra load on the back) in correlation with different angles of inclination and length of straps securing the kit. Overloads were applied to the knee, thigh and hip muscles [36].

Lesniak et al. also noted the impact of respiratory protective equipment on a firefighter’s job performance. Protective equipment makes it more difficult to perform firefighting tasks and increases the physical demands (effort) on the firefighter. In turn, the increased effort increases the risk of injury [37]. Our study found relationship between the number of injuries and the type of firefighting activities (Table 1). Personal protective equipment places an additional burden, restrains movements and increases dimensions but is necessary in the danger zone (fire, physical and chemical). Among the frequent circumstances of injury in the category where fire appeared, heavy smoke, limited visibility and making the firefighting line were noteworthy.

The Walker et al. study provides information on firefighting activities that require intense exertion in extreme environmental conditions; firefighters wearing impermeable, heavy and restrictive personal protective equipment are at risk of dehydration. This study confirms our results that the dynamics and conditions of a fire incident generate more injuries [38].

Firefighters’ duties include the implementation of emergency medical procedures at the level of qualified first aid (QFA). In the event of a firefighter accident, assistance is provided immediately directly at the scene by other firefighters. Medical kits are on the equipment of all service vehicles. Among the procedures, there are dressing wounds, attending burns and securing bone–joint injuries (immobilization). Firefighters follow framework procedures developed from international guidelines and standards [39,40,41,42].

Based on the analysis of the available literature, it can be concluded that this issue is not attractive for research interest, but its social significance and impact on the safety of not only those who rescue but also those for whom they serve is crucial from a practical perspective. Future research and analyses should focus not only on the epidemiology of firefighter eye injuries but also on the search for effective solutions for their prevention.

In our analysis, a significant part of injuries is related to sports. Physical education classes during the firefighter’s service are aimed at preparing for the performance of tasks, improving psychomotor skills and preparing for work requiring maximum physical effort (moving equipment, people and thermal stress). Sports in the fire service is an element of health prevention. The literature on sports injury prevention programs describes how to adapt them to the firefighter population and other professions with a similar level of risk [43,44].

## 5. Limitations

Major limitations of the study:

1. The lack of access to the firefighters’ medical records after the resulting injury (treatment procedures, diagnostic imaging, surgical treatment and medical diagnoses);
2. The lack of the age of the injured firefighters, allowing statistical calculations: the correlation of injury with age;
3. No information on the firefighters’ positions, which would allow us to determine the statistical probability of injury in correlation with the occupation: office work, driver and commander;
4. No information on possible permanent injury and impact on the course of further service;
5. In some of the post-injury reports collected, clinical data on the code according to the International Statistical Classification of Diseases and Related Health (ICD-10) were available, but the OOSH reports are not medical records and codes on the location and type of injury are rare, so the authors deviated from statistical summaries using ICD-10 codes. The most frequent codes were as follows:
  S93—Dislocation, sprain and tear of joints and ligaments at the ankle joint;
  S83—Dislocation, sprain and tear of the joints and ligaments of the knee [45].
6. Only a part of reports described in detail the official role of the firefighter whose injury was included in the study; therefore, the authors refrained from statistical analysis of this variable. However, available data show that firefighters–rescuers were more likely to have accidents than drivers and commanders;
7. Longitudinal studies to assess long-term injury effects and recovery patterns are impossible because of the anonymous nature of the population, the large number of people included in the study and the lack of possibility to observe the same people whether there is a repeatability of injuries;
8. The analysis may underestimate minor injuries that were not reported by firefighters.

## 6. Conclusions

A large number of musculoskeletal injuries are associated with sports activities, which is inherent in the risks of the activities. There is an increasing trend in sports-related injuries (percentage dimension). Despite these injuries, sports in the practice of firefighting are mandatory. The lower extremities are most affected by injuries during sports activities. These activities should be subjected to secondary analysis, taking into account changes: a better state of sports infrastructure. Technical-and-maintenance work should be carried out in additional security, with the participation of a second firefighter from the unit. Fires generate a large number of injuries, which can be related to the dynamics of the accident, difficult terrain, limited visibility and the burden of individual protective measures. The spring period dominates in the season category, which may be related to the difficulty of the terrain and return to increased sports activity after the winter period. In the firefighter education system and in the training courses, it seems reasonable to introduce content related to sport and work ergonomics.

The reporting of firefighter injuries and future analyses can address the gaps in the current state, such as addressing data gaps (the time elapsed since the start of service affecting the level of firefighter fatigue) and exploring lateralization. Future studies should explore long-term health outcomes of musculoskeletal injuries and evaluate prevention strategies.

## 7. Future Research Directions

The risk of injuries should be assessed in the fire department by appropriate units (OOSH sections), conducting periodic training among firefighters, where accident mechanisms serve as an example. Training should concern safe behaviors, risk factors, proper preparation for sports activities, warm-up, sports shoes, appropriate clothing, infrastructure for exercises and the supervision of management staff over the implementation of activities. Analyses concerning the population of the entire country should be compared to create a risk catalog for a given country. Risk can be correlated with financial and demographic conditions.

## Figures and Tables

**Table 1:** Univariate comparison of observation year cause, type and injury location.

Year	2021	2022	2023	*p*
Injuries, N	707	589	648	
Employed, N (whole country)	30,249	30,550	30,408	
Injuries (‰) from all employed	0.23	0.19	0.21	0.302
All interventions in country, N	534,500	560,900	486,400	
Injuries (‰) from all interventions	1.32	1.05	1.33	0.079
Cause, n (%)				<0.001
				χ^2^ = 54.8, df = 8
Injury in interventions-total	187 (26)	158 (27)	110 (17)	<0.001
Fire	98 (52)	66 (42)	46 (42)	
LT	62 (33)	57 (36)	45 (41)	
Not specified *	27 (14)	35 (22)	19 (17)	
Sport	319 (45)	287 (49)	408 (63)	<0.001
Training	32 (5)	30 (5)	32 (5)	0.883
TM work	60 (8)	34 (6)	29 (5)	0.008
Other	109 (15)	80 (14)	69 (11)	0.034
Injury type, n (%)				0.012
				χ^2^ = 19.5, df = 8
Sprain	423 (60)	365 (62)	379 (59)	0.454
Fracture	134 (19)	103 (17)	105 (16)	0.413
Tear	23 (3)	15 (2)	37 (6)	0.090
Dislocation	50 (7)	51 (9)	72 (11)	0.032
Other	77 (11)	55 (9)	55 (9)	0.313
Injury location, n (%)				<0.001
				χ^2^ = 28.7, df = 8
Lower limb	532 (75)	441 (75)	527 (81)	0.008
Upper limb	118 (17)	91 (15)	90 (14)	0.360
Head	3 (0.4)	12 (2)	11 (2)	0.026
Body-back	41 (6)	28 (5)	15 (2)	0.003
Body-front	13 (2)	17 (3)	5 (1)	0.020

abbreviations: LT—local threats, TM—technical and maintenance; * Activities unspecified—the report did not include information on the type of activities or the injury occurred on the way: “to activities” and “return from activities”, so the type of hazard did not directly affect the injury.

**Table 2:** Univariate comparison of type, location and season if injury.

	Intervention	Sport	Other	Training	TM Work	*p*
Injury type	N = 455	N = 1014	N = 258	N = 94	N = 123	0.002
					χ^2^ = 36.8, df = 16	
Sprain	281 (62)	622 (61)	142 (55)	49 (52)	73 (59)	0.168
Fracture	77 (17)	163 (16)	52 (20)	21 (22)	29 (24)	0.115
Tear	9 (2)	57 (6)	6 (2)	0	3 (2)	<0.001
Dislocation	42 (9)	88 (9)	23 (9)	12 (13)	8 (7)	0.604
Other	46 (10)	84 (8)	35 (14)	12 (13)	10 (8)	0.084
Season	N = 455	N = 1014	N = 258	N = 94	N = 123	<0.001
					χ^2^ = 45.5, df = 12	
Spring	124 (27)	298 (29)	63 (24)	34 (36)	22 (18)	0.017
Summer	102 (22)	339 (33)	70 (27)	29 (31)	37 (30)	<0.001
Autumn	110 (24)	206 (20)	63 (24)	18 (19)	33 (27)	0.208
Winter	119 (26)	171 (17)	62 (24)	13 (14)	31 (25)	<0.001
Injury location	N = 455	N = 1014	N = 258	N = 94	N = 123	<0.001
					χ^2^ = 76.6, df = 16	
Lower limb	334 (73)	838 (83)	184 (71)	61 (65)	83 (67)	<0.001
Upper limb	68 (15)	135 (13)	46 (18)	22 (23)	28 (23)	0.006
Head	3 (1)	17 (2)	4 (2)	2 (2)	0	0.323
Body back	36 (8)	15 (1)	17 (7)	7 (7)	9 (7)	<0.001
Body front	14 (3)	9 (1)	7 (3)	2 (2)	3 (2)	0.032

abbreviations: TM—technical and maintenance.

## Data Availability

The data presented in this study are available on request from the corresponding author due to state legal regulations regarding sharing data regarding internal security regarding country defense or security in crisis activities and units performing the above tasks.
